# Complex multiple renal calculi: stone distribution, pelvicalyceal anatomy and site of puncture as predictors of PCNL outcome

**DOI:** 10.1186/s40064-016-3017-4

**Published:** 2016-08-17

**Authors:** Amit Verma, Vinay Tomar, Shersingh Yadav

**Affiliations:** Urology Department, SMS Medical College, Jaipur, Rajasthan 302004 India

**Keywords:** Requirement of multiple tracts, Incidence of residual calculus, Timely multiple punctures, Haemoglobin fall, Creatinine rise

## Abstract

**Purpose:**

Management of patients with complex multiple renal calculi has always remained challenging and they pose many difficulties during percutaneous nephrolithotomy (PCNL) like higher incidence of residual calculus and multiple tracts requirement. The aim of our study was to evaluate the impact of pelvicalyceal system (PCS) anatomy, stone distribution and the site of puncture on the outcome of PCNL in patients with complex multiple renal calculi.

**Materials and methods:**

One hundred and ten patients with complex multiple renal calculi undergoing PCNL during January 2015 to December 2015 were enrolled in our study. Pelvicalyceal anatomy and the stone distribution were determined from intravenous urography. PCNL was done using standard technique. We evaluated the impact of PCS anatomy, stone distribution and the site of puncture on the surgical outcome.

**Results:**

Of all the studied pelvicalyceal anatomy variables, infundibular width, intercalyceal angle and PCS surface area affected the number of punctures. Stone distribution involving all the three calyces or middle and lower calyces was most unfavourable for achieving complete stone clearance. The middle calyceal puncture was almost equally good as the upper calyceal puncture in achieving stone clearance. With timely multiple punctures done, there was neither significant haemoglobin fall nor creatinine rise.

**Conclusion:**

Pelvicalyceal anatomy, stone distribution and site of puncture impacts the number of punctures required and stone clearance achieved in patients with complex multiple renal calculi undergoing PCNL. Based on these parameters we can predict which patient has a high likelihood of requirement of multiple punctures. With timely multiple punctures done, there is neither significant haemoglobin fall nor creatinine rise.

## Background

 Multiple stones in different parts of pelvicalyceal system (PCS) are considered as complex multiple renal calculi (Lee et al. 2014). Management of patients with complex multiple renal calculi has always remained challenging. Percutaneous nephrolithotomy (PCNL) is considered the gold standard first line treatment in the management of renal stones larger than 2 cm. Patients with complex multiple renal calculi pose many difficulties during PCNL like higher incidence of residual calculus and multiple tracts requirement. Creation of multiple percutaneous tracts has the potential risk of bleeding and higher complication rates compared with procedures using single tracts (Kukreja et al. 2004). The success of PCNL is highly related to optimal renal access. We evaluated impact of pelvicalyceal anatomy, stone distribution and site of a puncture on the requirement of multiple tracts and the incidence of residual calculus. We further evaluated the difference in the surgical outcome of the patients with single puncture and those with multiple tracts in terms of operative time, haemoglobin fall, creatinine rise, analgesic requirement, hospital stay and complications.

## Methods

One hundred and ten patients with complex multiple renal calculi undergoing PCNL in our institute during January 2015 to December 2015 were enrolled in our study. The study was approved by the ethics committee of our institute. Patients with coagulopathy and renal insufficiency were excluded from the study.

Pelvicalyceal anatomic factors including infundibulopelvic angle, intercalyceal angle, infundibular length and infundibular width, PCS surface area and the stone distribution were determined using standard sized intravenous urography (IVU) films.

**Fig. 1:**
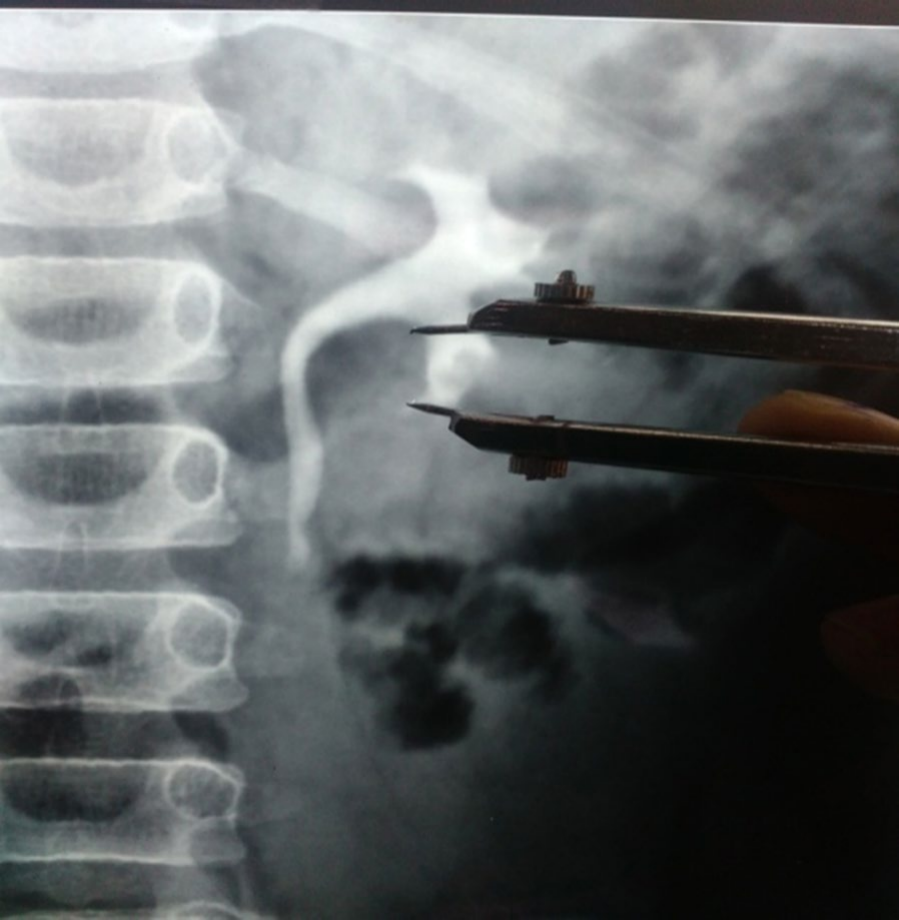
Measurement of infundibular length
- *Infundibular width* The infundibular width of the calyx was measured at the narrowest point along the infundibular axis as shown in Fig. 2.

Measurement of pelvicalyceal anatomic variables (Binbay et al. 2011):

- *Infundibular length* Infundibular length was measured as the distance between distal most point of the calyx and the pelvic-infundibular junction as shown in Fig. 1.

**Fig. 2:**
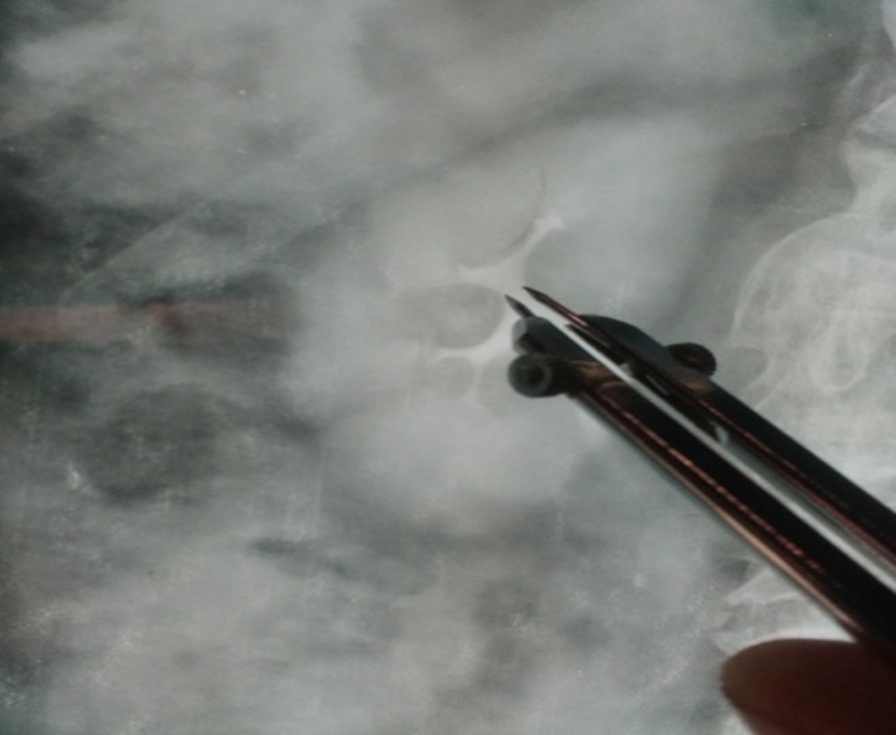
Measurement of infundibular width
- *Infundibulopelvic angle* The infundibulopelvic angle was measured as the angle formed at the intersection of the ureteropelvic axis and the central axis of that calyceal infundibulum as shown in Fig. 3.

**Fig. 3:**
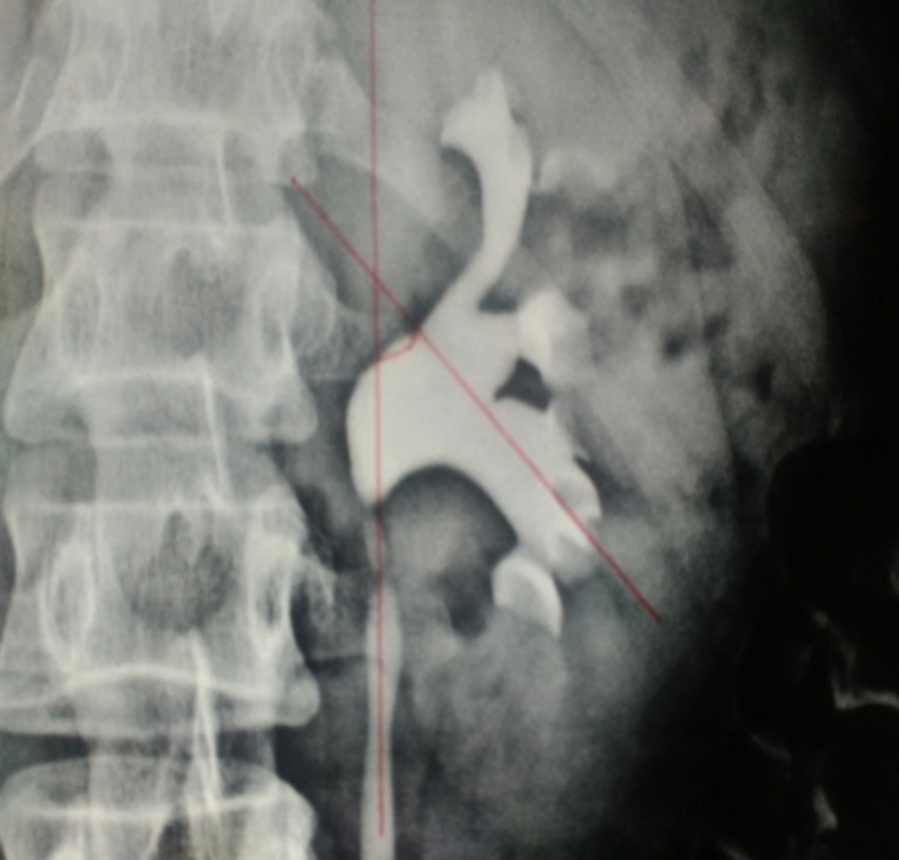
Measurement of infundibulopelvic angle
- *Intercalyceal angle* Intercalyceal angle was measured as the angle between the central axis of respective calyceal infundibulums as shown in Fig. 4.

**Fig. 4:**
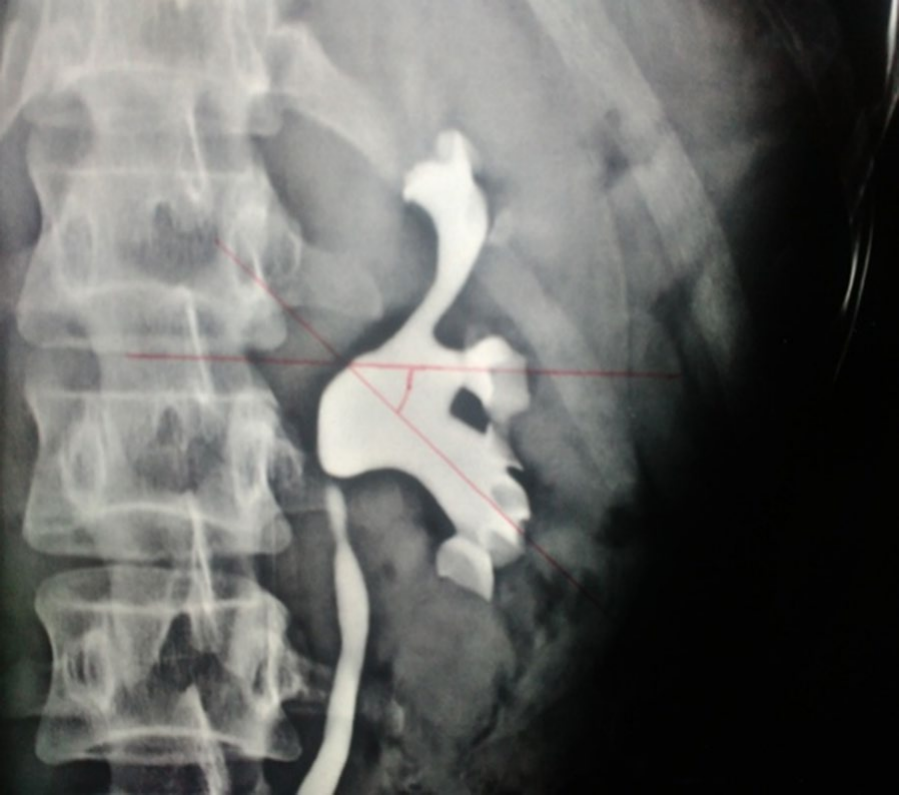
Measurement of intercalyceal angle
- *Pelvicalyceal system surface area* The surface area of the renal collecting system was measured using a 1-mm^2^ grid from the IVU.

Preoperative haemoglobin and serum creatinine were done.

PCNL was done using standard technique in prone position under general anesthesia using 26 Fr rigid nephroscope. The desired calyx for access was chosen with the criteria that it should have reasonable dilatation and maximum stone clearance can be achieved through that calyx. Maximum stone clearance with that tract was achieved. If required, multiple tracts were made in the favor of the patient and every event was recorded. Another calyx for access was chosen with the same criteria as described. Operative time was noted (starting from induction of anesthesia to completion of the procedure). Any complication or untoward event if occurred was noted.

Postoperative analgesic used was diclofenac intramuscularly and was given only when patient complained severe pain. On the first postoperative day, haemoglobin and serum creatinine were done. Postoperative X ray was done to look for any residual stone.

PCNL outcome was determined in terms of number of tracts, residual stone, operative time, haemoglobin fall, creatinine rise, analgesic required and hospital stay.

Data was collected and Statistical analyses were performed using the Statistical Package for the Social Science Version (SPSS) applying appropriate statistical tests.

## Results

For determining the impact of pelvicalyceal anatomy in determining the stone clearance, the data were stratified into two groups. In the 1st group were included when through a calyceal puncture, stone clearance was achieved in a different part of the PCS (apart from that calyx). In group 2 were included when another puncture was required to achieve stone clearance in a different part of the PCS. Mean infundibular width in group 1 was 5.9 mm while in group 2 it was 4.41 mm. Thus wide infundibula significantly favoured achieving stone clearance in different part of the PCS (p value .000375). Mean intercalyceal angle in group 1 was 78.49° while in group 2 it was 63.42°. Thus the wide intercalyceal angle significantly favoured achieving stone clearance in different part of the PCS (p value .010). The mean PCS surface area in group 1 was 109.9 mm^2^, while in group 2 it was 134.27 mm^2^. Hence group 2 had significantly large PCS surface area (p value .0209). There was no significant difference in mean infundibular length and the infundibulopelvic angle in two groups. Table 1 shows the PCS anatomy variables measurements in two groups.

**Table 1:** PCS anatomy variables measurements in two groups

	Group 1	Group 2	p value
Mean infundibular length (mm)	23.11	25.61	.1356
Mean infundibular width (mm)	5.9	4.41	.000375
Mean infundibulopelvic angle (°)	98.04	86.67	.617
Mean intercalyceal angle (°)	78.49	63.42	.010
Mean PCS surface area in (mm^2^)	109.9	134.27	.0209

**Fig. 5:**
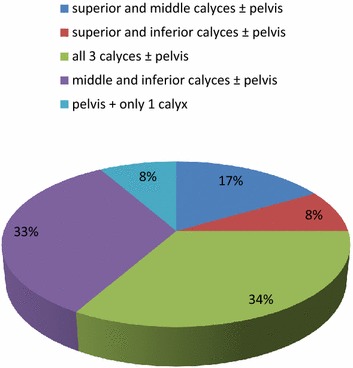
Pie chart showing requirement of multiple tracts according to stone distribution

In our study, approximately 33 % of patients with complex multiple calculus required more than one tract and the requirement of multiple tracts was affected by the stone distribution. The stone distribution most unfavorable for single tract was that involving all the three calyces and almost equally unfavorable was the stone distribution involving middle and lower calyces. Of the patients who required multiple tracts, approx. 34 % had involvement of all the three calyces and about 33 % had involvement of middle and lower calyces. Requirement of multiple tracts according to stone distribution is shown in Fig. 5.

**Fig. 6:**
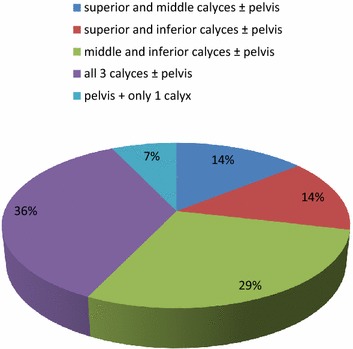
Pie chart showing residual calculus according to stone distribution

In our study, approximately 13 % of patients had residual calculus. Of the patients with residual calculus most unfavorable stone distribution was that with all three calyces (approx. 36 %) and next was those with stone distribution involving middle and lower calyces (approx. 29 %). Residual calculus according to stone distribution is shown in Fig. 6.

In our study, clearance of stone was affected by the puncture site. Approx. 55 % of patients achieved complete clearance with single puncture. Of these, 38 % had an upper calyceal puncture, 37 % had a middle calyceal puncture and 25 % had a lower calyceal puncture.

Surgical outcome between patients with multiple tracts and single tract was compared in terms of operative time, haemoglobin fall, creatinine rise, analgesic required and hospital stay. Patients requiring multiple punctures had significantly longer mean operative time (69.46 vs 51.68 min, p value < .01) and mean analgesic dose requirement (4.8 vs 3.53, p value < .01) while there was no significant difference in haemoglobin fall, creatinine rise and hospital stay. Details are shown in Table 2.

**Table 2:** Surgical outcome in patients with multiple tracts and single tract

	Multiple tract	Single tract	p value
Mean operative time (min)	69.46	51.68	<.00001
Mean hemoglobin fall (gm/dl)	1.75	1.67	.803
Mean creatinine rise (mg/dl)	.20	.18	.8235
Mean analgesic dose	4.8	3.53	<.00001
Mean hospital stay in days	3.11	3.08	.564
Blood transfusion requirement	4	3	.155
Postoperative fever	3	4	.55

Approx. 20 % of the patients required supracostal approach, but none of them developed any thoracic complications. There was no significant difference in complication rate between patients with single tract and those with multiple tracts.

## Discussion

PCNL is considered the first line recommended procedure for renal calculus. But patients with complex multiple renal calculi pose a special challenge for PCNL as these patients have high chances of incomplete stone clearance.

Effects of pelvicalyceal anatomy on achieving stone clearance in these patients have not yet been properly analyzed. In our study, of all the studied variables (infundibular length, infundibular width, infundibulopelvic angle, intercalyceal angle and PCS surface area), infundibular width, intercalyceal angle and PCS surface area significantly affected the stone clearance. Our study showed that if infundibular width is narrow, intercalyceal angle is more acute and the PCS surface area is larger, then approaching other calyces becomes difficult particularly if standard sized instruments were used, as in our study.

In a previous study conducted by Binbay et al. (2011), only PCS surface area significantly impacted the PCNL success rate while infundibular width and intercalyceal angle did not affect the PCNL outcome. This could be because both simple and complex renal calculus were analyzed in that study.

There had been very limited studies predicting the impact of stone distribution on the requirement of multiple tracts and the incidence of residual calculus in patients with complex multiple calculi. The requirement of multiple tracts was maximum in those with all three calyces involved and was almost equally high in patients with middle and lower calyces involved. Also the incidence of residual calculus was highest in those with all three calyces involved and next came those involving middle and lower calyces. Thus, our study showed that stone distribution most unfavourable for single puncture complete clearance was that with the involvement of all three calyces and next came that involving middle and lower calyces and then those with upper and middle calyces involved.

In our study, both superior and middle calyceal puncture were favorable for achieving complete stone clearance. Studies conducted by Aron et al. (2004) and Netto et al. (2005) had shown better results with superior calyceal puncture. The upper calyceal approach is believed to favor good manipulations of the nephroscope and forceps within the pelvicalyceal system while the lower calyceal approach caused undue angulations, and torque. This difference is believed to be because of the straight tract of the upper infundibulum along the long axis of the kidney and the anatomical lie of the kidney over iliopsoas muscle that cause the upper pole positioned more posterior as compared with the lower pole and these two factors provide excellent visualization of the pelvicalyceal system when an approach is made through the upper calyx (Singh et al. 2015). But the disadvantage with the upper calyceal approach is that it is not always subcostal, and may necessitate supracostal puncture which may cause serious thoracic complications. The upper pole of each kidney lies anterior to the posterior portion of the 11th and 12th ribs and during exhalation the lower limit of the parietal pleura crosses these ribs obliquely, such that the lateral portions of these ribs are inferior and lateral to the lower limit of the pleura (Munver et al. 2001). The incidence of thoracic complication during supracostal punctures in various studies ranges between 3 and 16 % (Sukumar et al. 2008; Lojanapiwat and Prasopsuk 2006). Keeping this consideration in mind while choosing puncture site, the middle calyceal puncture was very common in our study and almost equally good results were achieved using middle calyceal puncture as the upper calyceal puncture.

In our study total only ten patients required supracostal approach, but none of the patient developed any thoracic complication. This could be possibly because in patients at risk for thoracic complications, we preferred middle calyceal puncture.

Although the safety of creating percutaneous renal tracts is well established (Alken 1984), there is still a concern about the use of multiple tracts for the treatment of complex renal calculi.

A study conducted by Fayad et al. (2014) had shown that PCNL with multiple tracts carries a significant risk of adversely affecting renal function. In our study, we found no significant difference in mean creatinine rise in patients with multiple tracts and in those with single tract. A study conducted by Hegarty and Desai (2006) had shown that patients with multiple tracts had a more significant rise in serum creatinine. A study conducted by Akman et al. (2010) similar to our study had shown that difference in mean rise in serum creatinine in patients with single puncture and those with multiple tracts was not significant.

In our study, there was no significant difference in fall in haemoglobin between two groups. Similar results were shown by Hegarty and Desai (2006) in their study.

In our study, patients with multiple tracts had significantly longer operative time. Similar results were shown in a study conducted by Akman et al. (2010).

In our study there was no significant difference in mean hospital stay among patients with single puncture and those with multiple tracts. While a study conducted by Hegarty and Desai (2006) had shown that patients with multiple tracts had significantly longer hospital stay. Also, our study showed that patients with multiple tracts had a more analgesic requirement than those with single tract.

## Limitations of our study

- Single centre study.
- Assessment of stone distribution was performed using IVU, which measures only two PCS planes.
- Standard adult size nephroscope was used.

## Conclusion

Pelvicalyceal anatomy, stone distribution and site of puncture impacts the number of punctures required and stone clearance achieved. Of all the studied pelvicalyceal anatomy variables, infundibular width, intercalyceal angle and PCS surface area affect the number of punctures required during PCNL in patients with complex multiple renal calculi. Narrow infundibular width, acute intercalyceal angle and large PCS surface area restrict in achieving complete stone clearance in different part of the PCS.

Stone distribution involving all the three calyces or middle and lower calyces is most unfavourable to achieving complete stone clearance. Middle calyceal puncture is almost equally good as the upper calyceal puncture in achieving stone clearance.

With timely multiple punctures done, there is neither significant haemoglobin fall nor creatinine rise though there are longer operative time and analgesic requirement. These conclusions are based on study using standard adult size rigid nephroscope. Additional studies should be designed to further validate our results.

## References

[CR1] Akman T, Sari E, Binbay M, Yuruk E, Tepeler A, Kaba M (2010). Comparison of outcomes after percutaneous nephrolithotomy of staghorn calculi in those with single and multiple accesses. J Endourol.

[CR2] Alken P (1984). Percutaneous nephrolithotomy. Urologe A.

[CR3] Aron M, Goel R, Kesarwani PK, Seth A, Gupta NP (2004). Upper pole access for complex lower pole renal calculi. BJU Int.

[CR4] Binbay M, Akman T, Ozgor F, Sari E, Erbin A, Kezer C (2011). Does pelvi-calyceal system anatomy affect success of percutaneous nephrolithotomy?. Urology.

[CR5] Fayad AS, Elsheikh MG, Mosharafa A, Sergany RE, Abdel-Rassoul MA, Elshenofy A (2014). Effect of multiple access tracts during percutaneous nephrolithotomy on renal function: evaluation of risk factors for renal function deterioration. J Endourol.

[CR6] Hegarty NJ, Desai MM (2006). Percutaneous nephrolithotomy requiring multiple tracts: comparison of morbidity with single-tract procedures. J Endourol.

[CR7] Kukreja R, Desai M, Patel S, Bapat S, Desai M (2004). Factors affecting blood loss during percutaneous nephrolithotomy: prospective study. J Endourol.

[CR8] Lee JW, Cho SY, Jeong CW, Yu J, Son H, Jeong H, Oh SJ, Kim HH, Lee SB (2014). Comparison of surgical outcomes between laparoscopic pyelolithotomy and percutaneous nephrolithotomy in patients with multiple renal stones in various parts of the pelvocalyceal system. J Laparoendosc Adv Surg Tech A.

[CR9] Lojanapiwat B, Prasopsuk S (2006). Upper-pole access for percutaneous nephrolithotomy: comparison of supracostal and infracostal approaches. J Endourol.

[CR10] Munver R, Delvecchio FC, Newman GE, Preminger GM (2001). Critical analysis of supracostal access for percutaneous renal surgery. J Urol.

[CR11] Netto NR, Ikonomidis J, Ikari O, Claro JA (2005). Comparative study of percutaneous access for staghorn calculi. Urology.

[CR12] Singh R, Kankalia SP, Sabale V, Satav V, Mane D, Mulay A (2015). Comparative evaluation of upper versus lower calyceal approach in percutaneous nephrolithotomy for managing complex renal calculi. Urol Ann.

[CR13] Sukumar S, Nair B, Ginil KP, Sanjeevan KV, Sanjay BH (2008). Supracostal access for percutaneous nephrolithotomy: less morbid, more effective. Int Urol Nephrol.

